# *Lactobacillus plantarum* and *Lactobacillus acidophilus* enhance growth performance, immunity, cecal microbiota, and vital organs histomorphology in rabbits

**DOI:** 10.1038/s41598-025-33763-4

**Published:** 2026-01-23

**Authors:** Hitham Anas, Mahmmoud A. A. Mohamed, Rasha I. M. Hassan, Walaa M.S. Gomaa, Fatma El-Zahraa A. Mustafa

**Affiliations:** 1 Animal Production Department, Faculty of Agriculture, Al-Azhar University, Assiut Branch, Al Wilidiyyah, Assiut, 71524 Egypt; 2 Department of Nutrition and Clinical Nutrition, Faculty of Veterinary Medicine, Assiut University, Assiut, 71515 Egypt; 3 Department of Cell and Tissues, Faculty of Veterinary Medicine, Assiut University, Assiut, 71515 Egypt

**Keywords:** Probiotics, Growing rabbits, Performance, TNF-α, Synaptophysin, Histomorphometry, Diseases, Gastroenterology, Immunology, Microbiology, Physiology, Zoology

## Abstract

This study aimed to examine how adding two levels of probiotics, *Lactobacillus plantarum* and *Lactobacillus acidophilus*, to the growing V-line rabbits’ basal diet affects growth performance, carcass parameters, hematology, serum biochemistry, digestibility, cecal microbiota, economic evaluation of the diet, and histological and immunohistochemical features of the intestine, kidneys, liver, and heart. Sixty healthy five-week-old male rabbits were allocated at random to three groups, each with four replicates of five rabbits. The standard basal diet was provided to the three groups, with probiotics added to the second and third groups at 0.25 g/kg and 0.50 g/kg, respectively, for the 56-day experimental period. Probiotic supplementation significantly (*P* < 0.05) improved growth parameters and the weights of internal organs, while reducing the percentage of abdominal fat. White blood cell counts and other hematological parameters increased significantly (*P* < 0.05). Rabbits supplemented with 0.25 and 0.50 g/kg of probiotics showed significantly (*P* < 0.05) higher serum total protein, globulin, albumin, T3, T4, IgM, IgG, IgA and levels, and significantly (*P* < 0.05) lower triglycerides, ALT, and AST levels compared with the control one. Probiotic supplementation increased (*P* < 0.05) the digestibility of dry matter, organic matter, crude fiber, nitrogen-free extract, crude protein, and ether extract. It positively influenced beneficial cecal microbiota. Histological data showed increased villus length, crypt depth (CD), and epithelial thickness in the intestines. The kidney’s renal corpuscle and glomeruli diameter, along with CMFs diameter, increased. Liver PAS staining showed a dose-dependent increase. TNF-α expression rose significantly in both the small and large intestines, while synaptophysin increased in the large intestine (LI). Therefore, adding probiotics to the rabbit diet could improve performance, hematology, serum biochemistry, nutrient digestibility, cecal microbiota, and the economic evaluation of the diet, as well as the histological features of the intestine, kidneys, liver, and heart.

## Introduction

Food-feed competition, global warming, and deforestation increase pressure on the livestock industry, especially in developing nations struggling with food^1,2^. Domestic rabbits (Oryctolagus cuniculus), with their short life cycle, high feed efficiency, quick gestation, and productivity, offer a sustainable solution without competing with humans for food^3,4^.

Rabbits are hindgut fermenters and herbivores that exhibit a unique form of digestion called cecotrophy^5^. This process involves recycling low-quality proteins through cecal fermentation, resulting in cecotrophs that supply essential nutrients^6^.

This results in healthy, nutrient-rich meat that is low in cholesterol, rich in protein, and high in polyunsaturated fatty acids^7^. Rabbit production can thus boost the economy, mainly through meat, with fur used in various industries^8,9^. Unfortunately, rabbits often suffer gut imbalance, causing digestive issues like enteritis, which can be fatal due to diarrhea and dehydration^10^. Addressing this is vital for gut health. Feed additives, such as probiotics, can help resolve these issues and improve growth, immunity, and overall health^5^.

Probiotics are active microorganisms used in feed and food for their safety and sustainability^11^. While antibiotics at sub-therapeutic levels can enhance animal performance, they pose public health risks from resistant bacteria^12^. Probiotics offer a safe alternative, primarily consisting of Gram-positive bacteria, but may also include fungi, yeast, or Gram-negative bacteria^13^. *Lactobacillus plantarum*, a common probiotic in animal diets, benefits metabolism^14^. Its effects stem from its antioxidant properties, gut barrier support, and the boosting of beneficial microbes^15^. In rabbits, it enhanced body weight gain, feed efficiency, and gut health by reducing *E. coli*^16^. *Lactobacillus acidophilus*, another probiotic studied in animals, positively influences immunoglobulin production, reduces diarrhea and digestive issues, and lowers rabbit mortality^17^. A recent study found that supplementing rabbit diets with heat-treated *Lactobacillus acidophilus* improved protein and fiber digestibility, cecal fermentation, and performance^18^.

Through immunological reaction, neuroendocrine communication, and nutritional absorption, the gut is essential for preserving bodily homeostasis^1–3^. Probiotics influence the body through various mechanisms, one of which is their effect on the intestine, where they strengthen the epithelial barrier, regulate the enteric nervous system, and modulate the production process of pro-inflammatory cytokines^4–6^. Histology and immunohistochemistry, as essential tools, provide valuable insights into these mechanisms. Numerous research studies have demonstrated how probiotics improve gut structure^7–10^.

According to the available literature, this appears to be the first investigation assessing the dose-dependent responses to a probiotic mixture on the micromorphological features of the intestine and several vital organs in rabbits. Additionally, novel insights into assessing intestinal immunity and neuroendocrine activity through immunohistochemical markers.

The purpose of this study was to investigate the effects of introducing two distinct concentrations of *Lactobacillus acidophilus* and *Lactobacillus plantarum* as a novel strategy in rabbit diets on performance, carcass traits, hematology, blood metabolites, immunity, antioxidation, digestibility, cecal microbiota, and the histological features of the intestine, liver, kidney, and heart, with particular focus on immune and neuroendocrine markers in the intestine.

## Materials and methods

Animal treatment and management during the experiment strictly adhered to the ethical standards and guidelines established by the Faculty of Veterinary Medicine Ethics Committee at Assiut University in Egypt (06/2025/0353).

### Probiotic description

Two probiotic supplements in a powder form available commercially from Phytobiochem, Egypt, were used in our study: *Lactobacillus plantarum* (Phytobiochem, Egypt) and *Lactobacillus acidophilus* (Phytobiochem, Egypt). Two different dosages of *Lactobacillus plantarum* and *Lactobacillus acidophilus* were administered in the experiment: 0.25 g/kg and 0.50 g/kg of diet, based on earlier studies^16,19,20^.

### Animal management and housing

A private rabbit farm in Manfalut, Assiut, Egypt, was used for the experiment. Sixty healthy male V-line five-week-old growing rabbits, weighing approximately 785 g, were selected to conduct the study. The animals were examined for the presence of external parasites or any skin conditions. A 50 × 50 × 40 cm galvanized wire cage was used for each rabbit. They provided ad libitum feed and water, with stainless steel feeders and drinking nipples supplied to all cages. The management and environmental circumstances for each rabbit were the same. The experiment’s temperature and humidity ranged from 20 to 30 °C and 55% to 63%, respectively. The light cycle was set to 16 h of light and 8 h of darkness.

### Experimental design and diets

A computer-generated random number sequence was used to randomly assign the sixty rabbits into three treatment groups of 20 rabbits each. Each treatment was subdivided into four replicates, with five rabbits in each. The three treatment groups received a standard basal diet formulated based on previous studies^11,21^ to satisfy the growing rabbits’ nutritional needs. Probiotics were added to the second and third groups: the second group received 0.25 g/kg of *Lactobacillus plantarum* (15 × 10^9^ CFU/kg) combined with *Lactobacillus acidophilus* (20 × 10^9^ CFU/kg), while the third group was given 0.50 g/kg of *Lactobacillus plantarum* (15 × 10^9^ CFU/kg) along with *Lactobacillus acidophilus* (20 × 10^9^ CFU/kg). Between the ages of 5 and 13 weeks, the experiment lasted for eight weeks. The usual basal rabbit diet’s chemical and physical nature is presented in Table 1.

**Table 1:** Ingredients and chemical composition of the basal diet.

Item	Basal diet*
**Ingredients (g/kg)**	
Yellow corn^1^	100.00
Soybean meal^2^	160.00
Wheat bran^3^	100.00
Berseem hay^4^	550.00
Molasses^5^	50.00
Vegetable oil^6^	30.00
Salt	5.00
Premix^7^	5.00
**Chemical composition (%)**	
Crude protein	17.93
Ether extract	5.31
Crude fiber	14.79
Calcium	0.82
Total phosphorus	0.41
DE, Kcal/Kg diet^8^	2488

* The basal diet was fed to three groups of rabbits, with the probiotics’ mixture (*Lactobacillus plantarum* + *Lactobacillus acidophilus*) added to the 2nd and 3rd groups at 0.25 & 0.5 g/kg, respectively.

^1^Composition (as fed basis): 8.75% CP, 4.41% EE, 1.80% CF, and 3500 DE (Kcal/Kg diet).

^2^Composition (as fed basis): 44.80% CP, 2.78% EE, 6.10% CF, and 3160 DE (Kcal/Kg diet).

^3^Composition (as fed basis): 15.55% CP, 4.65% EE, 11.00% CF, and 2610 DE (Kcal/Kg diet).

^4^Composition (as fed basis): 14.80% CP, 1.80% EE, 22.80% CF, and 1780 DE (Kcal/Kg diet).

^5^Composition (as fed basis): 3.90% CP, 0.10% EE, and 2440 DE (Kcal/Kg diet).

^6^Composition (as fed basis): 99.00% EE, and 9000 DE (Kcal/Kg diet).

^7^Supplied per kilogram of dietary DM: Vit. A, 6,250,000 IU; Vit D3, 2,500,000 IU; Vit. E, 25,000 mg; Vit K3, 1750 mg; Vit B1, 500 mg; Vit B2, 2750 mg; Vit B6, 1250 mg; Vit B12, 10 mg; Nicotinic acid, 20,000 mg; Calcium pantothenate, 500 mg; Folic acid, 500 mg; Biotin, 50 mg; Iron, 22 g; Copper, 2.5 g; Zinc, 37.5 g; Manganese, 31 g; Iodine, 650 mg; Selenium, 113 mg; Cobalt, 50 mg.

^8^Digestible energy values of the ingredients were calculated according to NRC^101^.

### Performance measurements

Throughout the trial period, which spanned five to thirteen weeks, feed intake (FI) was tracked daily, each rabbit was weighed once a week, and the feed conversion ratio (FCR) and performance index (PI) were calculated. The PI was calculated by dividing the ultimate live body weight (kg) by the FCR and then multiplying the result by 100^22^.

### Carcass parameters

Six rabbits were randomly selected from each group to be humanely slaughtered when the study was completed. The live weights of the rabbits were noted as pre-slaughter weights after they had been fasted for 12 h with unlimited access to water. Following that, they were manually slaughtered in compliance with the World Rabbit Science Association’s regulations^23^. After allowing the rabbits to bleed and removing the gastrointestinal tract and skin, the weights of the hot carcasses were measured. The weight of abdominal fat and internal organs, including the heart, lungs, liver, kidneys and spleen was recorded.

### Hematological and serum biochemical parameters

The jugular veins of the same animals that were used for carcass measurements were used to extract blood samples. Each sample was split into two portions using vacuum tubes (Egyptian Trade CO, Cairo, Egypt): one for hematology tests, using sodium-heparinized vacuum tubes kept at 4 °C, and the other for serum biochemical analysis, using additive-free vacuum tubes. Before storage at -20 °C, serum analysis tubes were centrifuged (Millipore Sigma, USA) at 4000 rpm for 5 min^5^.

For hematology analysis, the following parameters were measured: Hemoglobin (HGB), Hematocrit (HCT), Mean Corpuscular Hemoglobin (MCH), White Blood Cells (WBCs, 10^3^), Monocytes (MON), Lymphocytes (LYM), and Neutrophils (NEU)^24^.

For serum biochemical analysis, including antioxidant biomarkers and immunoglobulin determination, the following parameters were measured spectrophotometry (Spectra Lab Scientific Inc., USA) using commercially available kits (EGY- CHEM, Badr City, Egypt) Total protein (TP), Alanine aminotransferase (ALT), Aspartate aminotransferase (AST), Globulin (GLO), Albumin (ALB), Triiodothyronine (T3), Thyroxine (T4), Cholesterol (CHO), Triglycerides (TRI), Immunoglobulin M (IgM), Immunoglobulin G (IgG), and Immunoglobulin A (IgA).

### Digestibility coefficients of nutrients

The digestibility trial commenced a week prior to the experimental end, when 15 rabbits (5 from each group) were haphazardly chosen and separately housed in metabolic cages to collect their feces. Feed consumption was documented on a daily basis, and fecal samples were collected 24 h after the daily feeding procedure and continued for six days. Nitrogen-free extract (NFE), ether extract (EE), organic matter (OM), dry matter (DM), crude protein (CP), and crude fiber (CF) digestion coefficients were measured^25^. The digestible crude protein (DCP) and total digestible nutrients (TDN) of the experimental diets were calculated to evaluate their nutritional value according to El-Kelawy et al.^26^. DCP was calculated based on the CP content and its digestibility coefficient. TDN was calculated by summing % digestible CP, % digestible fat, % digestible NFE, and % digestible CF (multiplied by 2.25 to account for its higher energy content).

### Cecal microbiota

Immediately following the slaughter of animals, cecal samples were meticulously separated, collected in sterile containers, maintained on ice, and subsequently stored at -20 °C and diluted 10 days after collection for subsequent analysis.

Total bacterial count (TBC) and selected microbial counts (*Salmonella*, *Lactobacillus*, and *Escherichia coli*) were determined using the serial dilution method (from 10^− 1^ to 10^− 8^) with plating 0.1 milliliters of each diluted sample 3 times on the appropriate agar medium (Nutrient agar (Oxoid Manual, 1965) was employed for the total bacterial count. *Lactobacillus* MRS agar (HiMedia M641) was used for cultivating *Lactobacillus* species. Salmonella-Shigella (SS) agar was employed for the selective isolation of *Salmonella* species, while *Escherichia coli* were enumerated and identified using Violet Red Bile Agar (VRBA, M049 – HiMedia Laboratories) and MacConkey Agar (Oxoid, UK) according to the manufacturer’s instructions)^27^.

### Histological and immunohistochemical examination

For histological studies, samples were collected from the small intestine (jejunum), large intestine (cecum), liver, kidney, and heart of slaughtered animals from different experimental groups. Samples were fixed in formalin (10%), dehydrated, cleared, and embedded. Then, samples blocks were cut (4–5 μm) and stained with HE satin and PAS stain Harris hematoxylin and eosin^28,29^.

Immunohistochemistry was performed on Sect. (5 μm thick) of the small and large intestine, as described previously^30–34^. Sections were washed in phosphate-buffered saline (pH 7.4) after being deparaffinized in xylene and rehydrated using graded ethanol. To stop the endogenous peroxidase activity, hydrogen peroxide was given to the sections. Following antigen retrieval, the slides were heated in sodium citrate buffer, allowed to cool to room temperature, and then cleaned with PBS. To reduce nonspecific binding, sections were incubated with an Ultra V block. The primary antibodies include rabbit polyclonal anti-TNF-α (Catalog No. A11534) and rabbit polyclonal anti-synaptophysin (Catalog No. A6344). Mayer’s hematoxylin is used as a counterstain.

Histological measurement of the stained section was carried out using ImageJ software, the following measurements were taken: Villi length in the small intestine (SI), thickness of the epithelium in the SI, CD in SI, thickness of the epithelium in the LI, CD in the LI, diameter of hepatocytes, diameter of hepatocyte nuclei, renal corpuscle diameter, glomerular diameter, diameter of CMF, diameter of CMF nuclei, area % intensity of PAS stain in the SI, area % intensity of PAS stain in the liver, area % intensity of TNF-α in the lamina propria (LP) of the SI, area % intensity of TNF-α in the LP of the LI, area % intensity of synaptophysin in the LP of the SI, and area % intensity of synaptophysin in the LP of the LI.

### Economic evaluation

The economic evaluation of the experiment was conducted by calculating several parameters: total feed costs (including the cost of ingredients and probiotics), the difference between revenue and costs is described as the net profit, economic efficiency (net profit multiplied by 100 and divided by total cost), and relative economic efficiency (economic efficiency of the group divided by that of the control, multiplied by 100)^35,36^.

### Statistical analysis

To assess the differences between the experimental treatments, growth performance, carcass parameters, hematology, biochemical serum analysis, digestibility trial, cecal microbiota, histological, and immunohistochemistry data were analyzed using the ANOVA test and Duncan’s multiple range test. Using SPSS 26.0 software, a difference is considered significant if the P value < 0.05. The data for each variable are presented as means accompanied by pooled standard errors.

## Results

### Growth performance and carcass parameters

Table 2 displays how dietary treatments affect the growing rabbits’ growth performance and carcass indices. When compared to the control diet, probiotic supplementation of rabbit diets increased (*P* < 0.05) the final BWG and BW and improved (*P* < 0.05) the FCR and PI. Probiotic-supplemented diets significantly (*P* < 0.05) reduced FI in rabbits. Furthermore, rabbits fed diets with 0.50 g of probiotics per kg of diet exhibited the best (*P* < 0.05) results in BW, BWG, FCR, and PI.

**Table 2:** Effect of dietary treatments on performance and carcass parameters in growing rabbits.

Item	Treatment*				
	T1	T2	T3up	SEM	*P*-value
**Performance Parameters**					
Initial LBW (5 wks.), g	788.70	785.85	787.20	1.40	0.361
Final LBW (13 wks.), g	1728.4^c^	1909.4^b^	2216.8^a^	15.23	< 0.001
BWG, g	939.75^c^	1123.6^b^	1429.6^a^	15.08	< 0.001
FI, g	4810.5^a^	4384.8^b^	3911.8^c^	20.35	< 0.001
FCR, g/g	5.12^a^	3.92^b^	2.75^c^	0.05	< 0.001
PI, %	33.81^c^	49.06^b^	81.31^a^	1.37	< 0.001
**Carcass Parameters (%)**					
Hot carcass	87.54	89.89	88.73	0.816	< 0.783
Heart	0.30^c^	0.34^b^	0.40^a^	0.006	< 0.001
Spleen	0.05^c^	0.09^b^	0.13^a^	0.003	< 0.001
Liver	3.59^b^	4.48^a^	4.68^a^	0.105	< 0.001
Lungs	0.35^b^	0.39^a^	0.40^a^	0.004	< 0.001
Kidneys	0.65	0.66	0.67	0.008	< 0.403
Abdominal fat	2.33^a^	1.60^b^	0.82^c^	0.06	< 0.001

*T1: Control group, T2& T3, Probiotics-supplemented groups at 0.25 & 0.5 g/kg, respectively.

LBW: Live Body Weight, BWG: Body Weight Gain, FI: Feed Intake, FCR: Feed Conversion Ratio, PI: Performance Index.

Means with different superscripts in the same row differ significantly (*P* < 0.05).

Abdominal fat was substantially reduced in probiotic-supplemented rabbits than in control rabbits, while the relative weights of the heart, spleen, liver, and lungs were significantly larger (*P* < 0.05). Dietary therapies, however, had no effect on kidney or hot carcass percentages.

### Hematological and serum biochemical parameters

Table 3 provides a summary of the hematological parameters. When probiotics were added to the rabbit diets, the values of WBCs, HGB, HCT, MCH, MON, LYM, and NEU were considerably higher (*P* < 0.05) than in the control group.

**Table 3:** Effect of dietary treatments on hematological parameters in growing rabbits.

Item	Treatment*				
	T1	T2	T3	SEM	*P*-value
WBCs, × 10 ^3^/µl	4.98^c^	6.82^b^	8.76^a^	0.29	< 0.001
HGB, g/dl	10.67^c^	12.75^b^	13.58^a^	0.22	< 0.001
HCT, %	34.23^c^	41.01^b^	46.38^a^	1.00	< 0.001
MCH, pg	31.75^c^	36.50^b^	40.75^a^	0.79	< 0.001
MON, %	3.00^c^	6.50^b^	10.00^a^	0.83	0.001
LYM, %	22.50^c^	33.25^b^	44.00^a^	1.41	< 0.001
NEU, %	49.50^c^	63.50^b^	80.25^a^	2.16	< 0.001

*T1: Control group, T2& T3, Probiotics-supplemented groups groups at 0.25 & 0.5 g/kg, respectively.

WBCs: White Blood Cells, HGB: Hemoglobin, HCT: Hematocrit, MCH: Mean Corpuscular Hemoglobin, MON: Monocytes, LYM: Lymphocyte, NEU: Neutrophil.

Means with different superscripts in the same row differ significantly (*P* < 0.05).

The effect of probiotic supplementation on the serum biochemical metabolites of growing rabbits is displayed in Table 4. Rabbits fed probiotic-supplemented diets showed greater (*P* < 0.05) levels of blood total protein, albumin, globulin, T3, T4, IgA, IgG, and IgM, and lower levels of TRI, ALT, and AST than the control group.

**Table 4:** Effect of dietary treatments on serum biochemical parameters in growing rabbits.

Item	Treatment*				
	T1	T2	T3	SEM	*P*-value
TP (g/dL)	5.32^c^	6.27^b^	7.72^a^	0.23	< 0.001
ALB (g/dL)	3.98^b^	4.44^b^	5.74^a^	0.19	< 0.001
GLO (g/dL)	1.33^b^	1.82^a^	1.98^a^	0.14	0.026
A/G ratio	3.20	2.44	2.93	0.29	0.238
TRI (mg/dL)	175.00^a^	149.75^b^	125.25^c^	2.87	< 0.001
CHO (mg/dL)	180.25^a^	165.00^b^	146.00^c^	2.19	< 0.001
ALT (IU/L)	44.35^a^	35.70^b^	24.77^c^	1.29	< 0.001
AST (IU/L)	52.65^a^	39.72^b^	30.10^c^	1.22	< 0.001
T3 (ng/dl)	79.74^c^	92.00^b^	109.27^a^	2.02	< 0.001
T4 (ug/dL)	5.57^c^	7.67^b^	8.93^a^	0.20	< 0.001
IgA (mg/dL)	92.22^c^	104.25^b^	123.75^a^	2.82	< 0.001
IgG (mg/dL)	497.50^c^	577.00^b^	628.25^a^	9.29	< 0.001
IgM (mg/dL)	99.50^c^	113.75^b^	138.25^a^	2.07	< 0.001

*T1: Control group, T2& T3, Probiotics-supplemented groups at 0.25 & 0.5 g/kg, respectively.

TP: Total protein, ALB: Albumin, GLO: Globulin, A/G ratio: Albumin/Globulin ratio, TR: Triglycerides, CHO: Cholesterol, ALT: Alanine aminotransferase, AST: Aspartate aminotransferase, T3: Triiodothyronine, T4: Thyroxine, IgA: Immunoglobulin A, IgG: Immunoglobulin G, IgM: Immunoglobulin M.

Means with different superscripts in the same row differ significantly (*P* < 0.05).

### Digestibility coefficients of nutrients

Nutrient digestibility influenced by the dietary adding of probiotics is shown in Table 5. Supplementing with 0.25–0.50 g of probiotics per kg of diet significantly improved the digestibility of DM, OM, CP, EE, CF, and NFE, and increased the percentages of TDN and DCP (*P* < 0.05) contrasting with the control group. Furthermore, their values were higher (*P* < 0.05) with 0.50 g probiotic supplementation compared to 0.25 g.

**Table 5:** Effect of dietary treatments on the digestibility coefficients of nutrients (%).

Item	Treatment*				
	T1	T2	T3	SEM	*P*-value
Dry matter	60.77^c^	65.72^b^	68.39^a^	0.47	< 0.001
Organic matter	62.01^c^	68.43^b^	71.57^a^	0.40	< 0.001
Crude protein	71.70^c^	77.65^b^	80.29^a^	0.45	< 0.001
Ether Extract	62.96^c^	69.12^b^	74.35^a^	0.45	< 0.001
Crude fiber	28.59^c^	36.42^b^	40.35^a^	0.41	< 0.001
Nitrogen-free extract	64.51^c^	69.82^b^	71.78^a^	0.55	< 0.001
TDN	58.86^c^	65.35^b^	68.39^a^	0.66	< 0.001
DCP	12.48^c^	13.90^b^	14.38^a^	0.08	< 0.001

*T1: Control group, T2& T3, Probiotics-supplemented groups at 0.25 & 0.5 g/kg, respectively.

TDN: Total Digestible Nutrients, DCP: Digestible Crude Protein.

Means with different superscripts in the same row differ significantly (*P* < 0.05).

### Cecal microbiota

Results from Table 6 demonstrated that giving probiotics to growing rabbits had an impact on their cecal microbiota. Rabbits fed diets with increasing probiotic levels (0.25 and 0.50 g/kg) had significantly lower total bacterial counts and *Salmonella* populations (*P* < 0.05), along with higher *Lactobacillus* sp. counts (*P* < 0.05) compared to the control. The populations of E. coli did not differ significantly between the groups.

**Table 6:** Effect of dietary treatments on cecal microbiota in growing rabbits.

Item	Treatment*				
	T1	T2	T3	SEM	*P*-value
TBC (x10^7^)	11.46^a^	11.29^b^	11.25^b^	0.04	0.014
*Lactobacilli* (x10^4^)	4.87^c^	5.49^b^	5.69^a^	0.05	< 0.001
*E. coli* (x10^4^)	5.31	5.01	4.93	0.15	0.230
*Salmonella* (x10^4^)	5.57^a^	4.99^b^	4.83^c^	0.04	< 0.001

*T1: Control group, T2& T3, Probiotics-supplemented groups at 0.25 & 0.5 g/kg, respectively.

TBC: Total Bacterial Count.

Means with different superscripts in the same row differ significantly (*P* < 0.05).

### Histological findings

The small intestine (SI), LI, liver, kidney, and heart sections of rabbits from different groups were evaluated histologically.

**Fig. 1:**
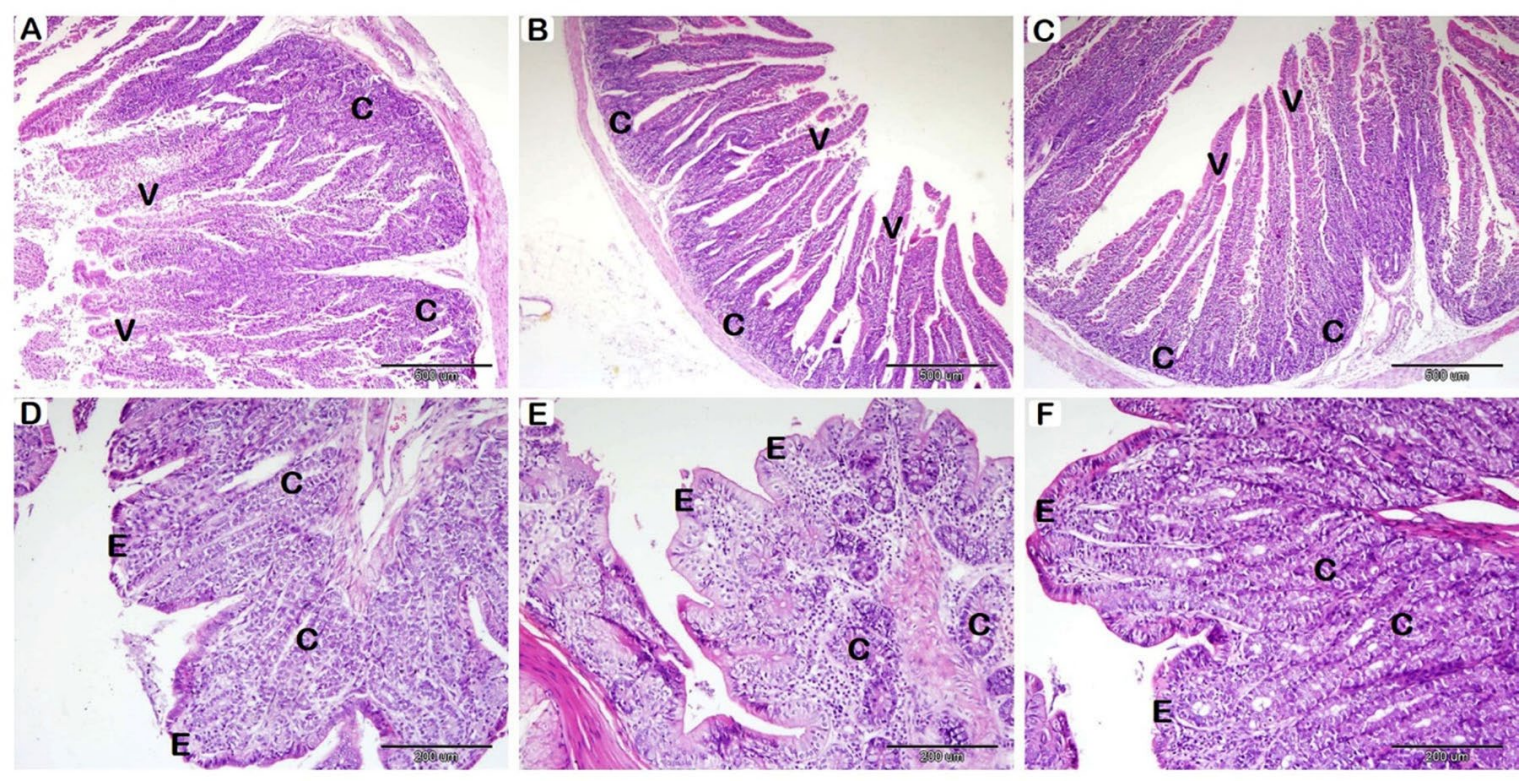
Histological examination of the rabbit SI (A-B-C), and LI (D-E-F), following probiotics supplementation with HE stain. (**A**): T1 group, (**B**): T2 group, and (**C**): T3 group. Villi (V); longer villi detected in T2 and T3. Crypts (**C**); the highest depth of crypt is detected in T3. (**D**): T1 group, (**E**): T2 group, and (**F**): T3 group. Epithelium (**E**); The Thickness of the epithelium is greater in T2 and T3. Crypts (C); the highest depth of crypt is detected in T3.

Histological data showed significant differences between groups across numerous parameters in the SI (Fig. 1A-B-C; Table 7). The control group exhibited significantly shorter villi; in contrast, probiotics-fortified groups showed a noticeable increase in villus length. However, the probiotic-supplemented groups did not differ significantly in villus length. Additionally, adding probiotics to the diet greatly improved the thickness of the intestinal epithelium. The intestinal epithelial thickness was lowest in the control group and highest in the 0.50 g/kg probiotic group and second in the 0.25 g/kg probiotic group. While there were no significant variations in intestinal CD between the control group and the 0.25 g/kg group, the depth of the intestinal crypts rose significantly in the 0.50 g/kg group when compared to the other two groups.

**Table 7:** Effect of dietary treatments on histological parameters in growing rabbits.

Item	Treatment*				
	T1	T2	T3	SEM	*P*-value
Villi length in the SI /um	845.03^b^	981.01^a^	973.66^a^	12.17	< 0.001
Thickness of the epithelium in the SI /um	97.96^c^	116.69^b^	147.37^a^	2.53	< 0.001
CD in the SI /um	166.98^b^	172.85^b^	221.46^a^	6.57	0.002
Thickness of the epithelium in the LI /um	18.89^b^	34.51^a^	31.97^a^	1.24	< 0.001
CD in the LI /um	444.35^b^	364.82^c^	683.13^a^	12.08	< 0.001
Diameter of hepatocyte/um	17.43	17.65	17.96	0.52	0.783
Diameter of hepatocyte nuclei/um	6.85	6.67	6.28	0.35	0.542
Renal corpuscle diameter/um	119.39^b^	88.88^c^	154.89^a^	4.25	< 0.001
Glomerular diameter/um	108.84^b^	86.03^c^	135.02^a^	2.82	< 0.001
Diameter of CMF/um	10.88^b^	14.76^a^	14.25^a^	0.45	0.002
Diameter of CMF nuclei/um	5.35	6.35	5.67	0.42	0.303

*T1: Control group, T2& T3, Probiotics-supplemented groups at 0.25 & 0.5 g/kg, respectively.

Means with different superscripts in the same row differ significantly (*P* < 0.05).

Histological assessments of the LI exposed statistically significant differences among the different groups (Fig. 1D-E-F, and Table 7). The thickness of the epithelium was significantly greater in probiotics-supplemented groups compared to the control. However, between probiotics-supplemented groups, there were no significant differences in epithelium thickness. In the group that received 0.50 g/kg of probiotics, a significant increase in CD was observed compared to the other two groups; the shallowest crypts were detected in the group that received 0.25 g/kg of probiotics.

**Fig. 2:**
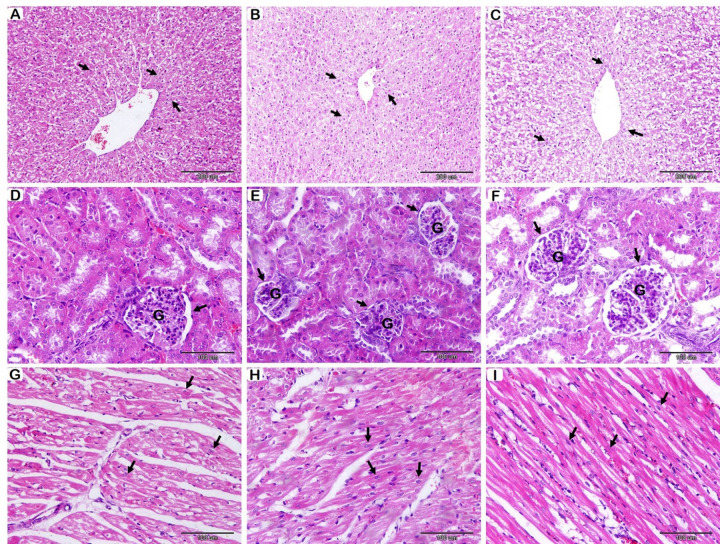
Histological examination of rabbit liver (A-B-C), kidney (D-E-F), and heart (G-H-I) following probiotics supplementation with HE stain. (**A**): T1 group, (**B**): T2 group, and (**C**): T3 group. Hepatocyte (arrows); no significant differences in the diameter of hepatocytes between different groups. (**D**): T1 group, (**E**): T2 group, and (**F**): T3 group. Renal corpuscle (arrows) and Glomerular (**G**) were significantly increased in T3. (**G**): T1 group, (**H**): T2 group, and (**I**): T3 group. Cardiac muscle fibres (arrows); the diameter of CMFs increased significantly in T2 and T3.

Histological evaluations of hepatic tissue revealed that the diameter of hepatocytes and the diameter of hepatocyte nuclei were statistically unaffected across different groups, indicating no significant differences. (Fig. 2A-B-C, and Table 7).

Renal histomorphometric data indicated significant differences among the different groups (Fig. 2D-E-F; Table 7). The diameter of the renal corpuscle differed significantly among groups, with the largest diameter observed in the group that received 0.50 g/kg of probiotics, followed by the control group, and then the group that received 0.25 g/kg of probiotics. Glomerular diameter showed a similar pattern, with the group that received 0.50 g/kg significantly larger in diameter than the other two groups.

Histomorphometric analysis of the cardiac muscle fibers (CMFs) confirmed a significant effect on fiber diameter, while nuclear diameter remained unchanged (Fig. 2G-H-I; Table 7). CMFs diameter increased significantly in probiotics-supplemented groups compared to the control group, with no significant difference between probiotics-supplemented groups. However, the nuclear diameter of CMFs showed non-significant differences between groups.

### Histochemical findings

**Fig. 3:**
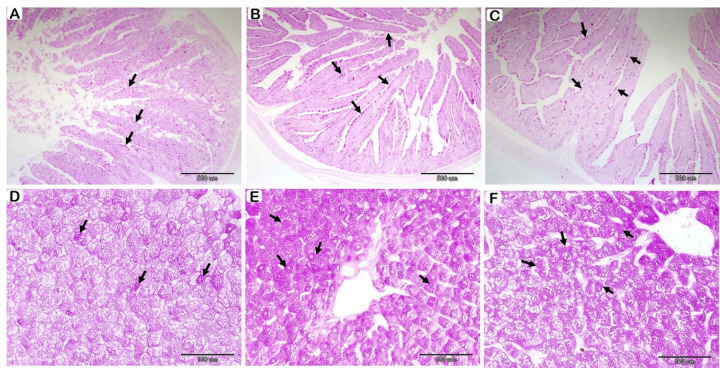
Histological examination of the rabbit SI (A-B-C) and liver (D-E-F) following probiotics supplementation with PAS stain. (**A**): T1 group, (**B**): T2 group, and (**C**): T3 group. Positive PAS staining (arrows); no significant difference between groups. (**D**): T1 group, (**E**): T2 group, and (**F**): T3 group. Positive PAS staining (arrows); increased significantly in a dose-dependent way.

PAS staining intensity in the SI statistically appeared non-significant between groups, but the higher mean values were detected in probiotics-supplemented groups compared to the control (Fig. 3A-B-C; Table 8). On the other hand, PAS staining affinity in hepatocytes increased significantly in a dose-dependent way; the group that received 0.50 g/kg displayed the highest affinity, followed by the group that received 0.25 g/kg and the control group.

**Table 8:** Effect of dietary treatments on histochemical parameters in growing rabbits.

Item	Treatment*				
	T1	T2	T3	SEM	*P*-value
Area % intensity of PAS stain in the SI	0.77	0.97	0.93	0.05	0.073
Area % intensity of PAS stain in the liver	14.29^c^	18.20^b^	22.65^a^	0.65	< 0.001

*T1: Control group, T2& T3, Probiotics-supplemented groups at 0.25 & 0.5 g/kg, respectively.

Means with different superscripts in the same row differ significantly (*P* < 0.05).

### Immunohistochemical analysis of TNF-α and synaptophysin expression

**Fig. 4:**
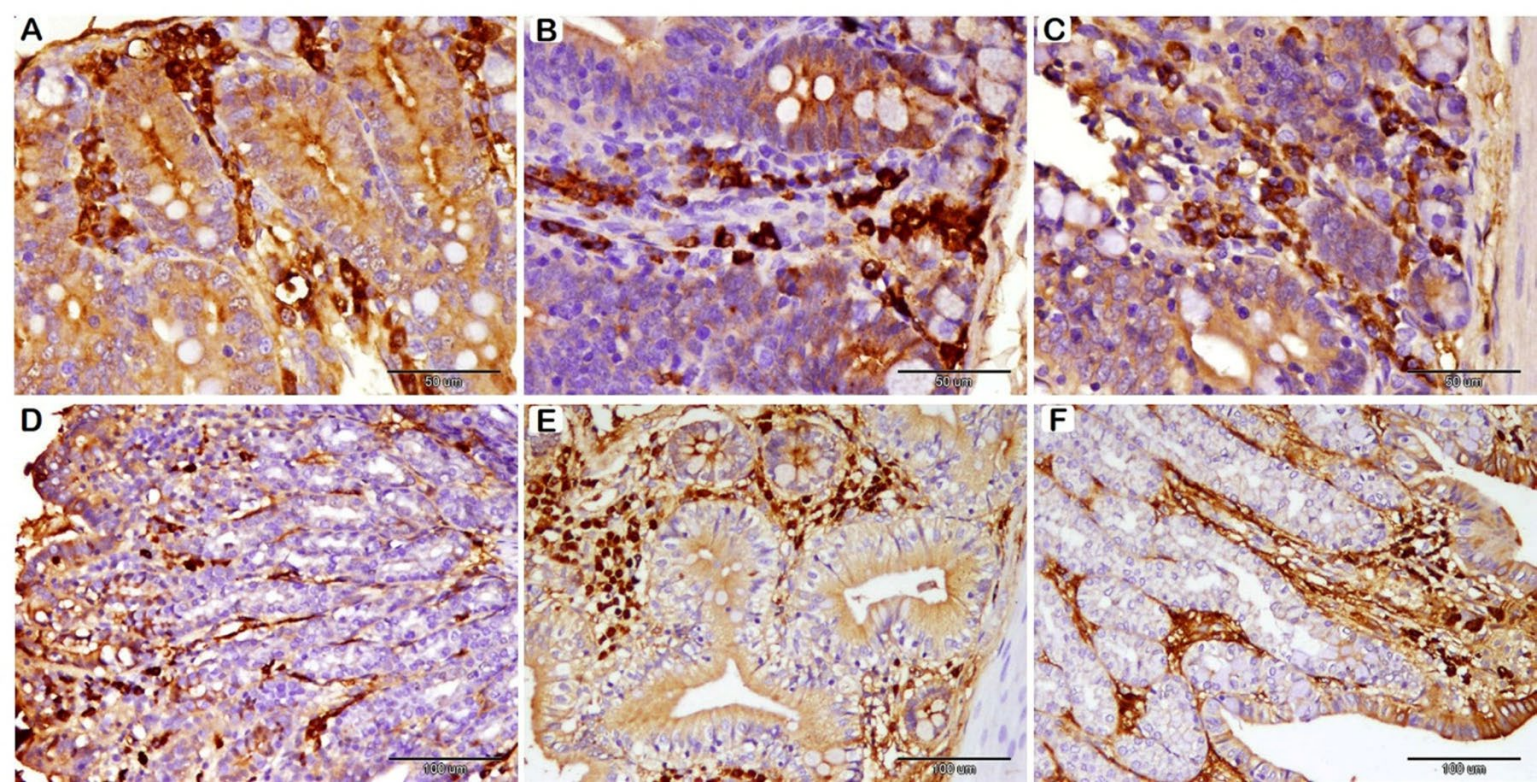
Immunohistochemical analysis of TNF-α in rabbit SI (A-B-C), and LI (D-E-F) following probiotics supplementation. (**A**): T1 group, (**B**): T2 group, and (**C**): T3 group; showed a significant dose-dependent rise in TNF-α Immunohistochemical expression (**D**): T1 group, (**E**): T2 group, and (**F**): T3 group; TNF-α expression in LI was significantly increased in T2 and T3.

**Fig. 5:**
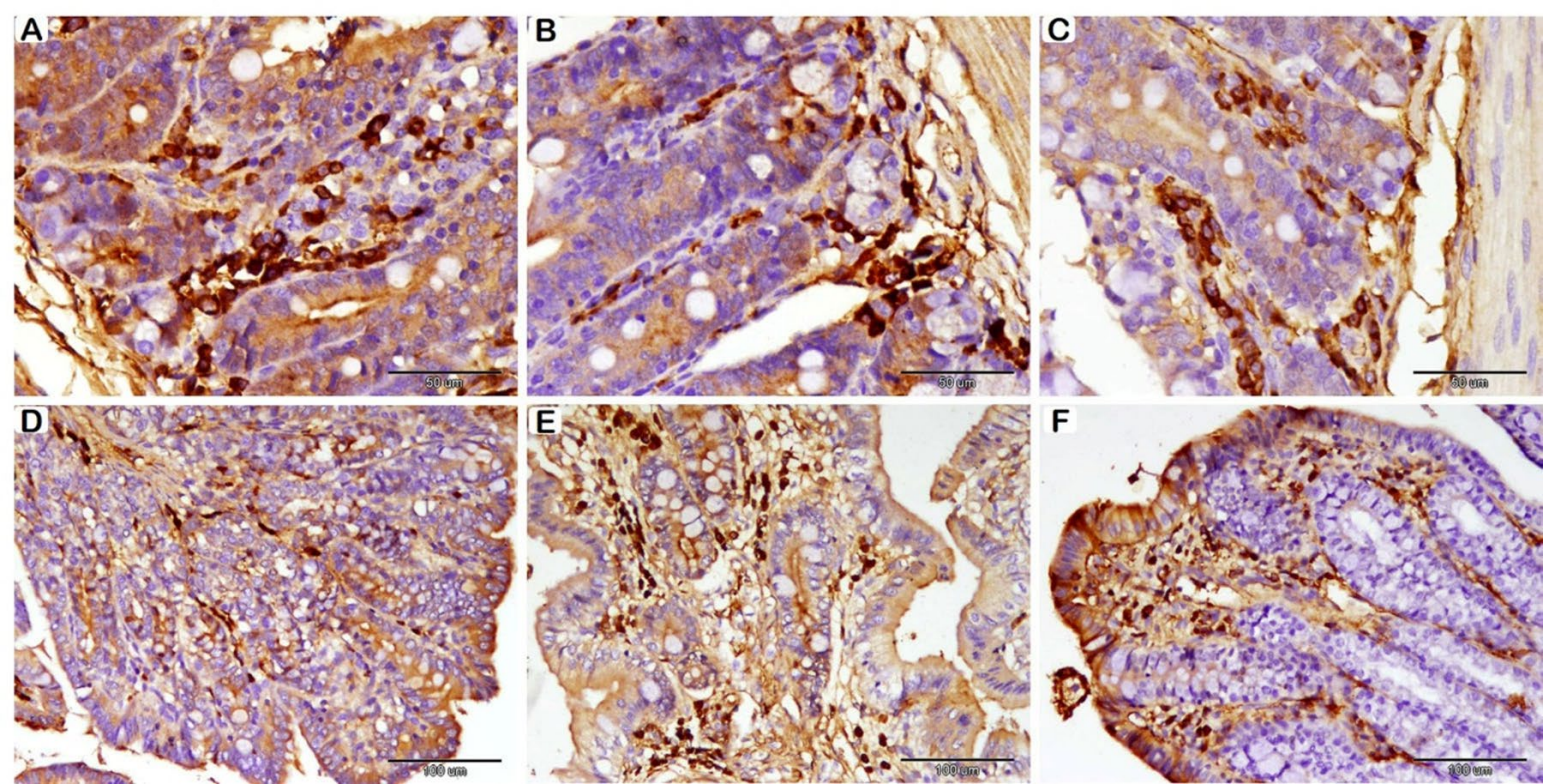
Immunohistochemical analysis of Synaptophysin in rabbit SI (A-B-C), and LI (D-E-F) following probiotics supplementation. (**A**): T1 group, (**B**): T2 group, and (**C**): T3 group; showed no significance difference between groups. (**D**): T1 group, (**E**): T2 group, and (**F**): T3 group; showed significantly increased expression in T2 and T3.

We next assessed the immunity and neuroendocrine enhancement of probiotic-supplemented groups by immunohistochemical staining for TNF-α and synaptophysin, respectively, in the SI and LI (Figs. 4 and 5; Table 9). TNF-α Immunohistochemical analysis of the SI indicated a significant dose-dependent rise in TNF-α Immunohistochemical expression across the groups, with 0.50 g/kg of probiotics showing the highest levels and the control group showing the lowest. Moreover, TNF-α expression in the LI was significantly increased in the probiotics-supplemented groups compared to the control. Synaptophysin immunohistochemical assessment in the SI showed no significant difference between the three groups. However, synaptophysin immunohistochemical analysis in the LI showed significantly increased expression in probiotics-supplemented groups compared to the control.

**Table 9:** Effect of dietary treatments on immunohistochemical parameters in growing rabbits.

Item	Treatment*				
	T1	T2	T3	SEM	*P*-value
Area % intensity of TNF-α in the LP of the SI	4.05^c^	6.39^b^	8.18^a^	0.15	< 0.001
Area % intensity of TNF-α in the LP of the LI	4.78^b^	7.37^a^	7.03^a^	0.41	0.008
Area % intensity of Synaptophysin in the LP of the SI	5.84	5.89	5.57	0.11	0.170
Area % intensity of Synaptophysin in the LP of the LI	2.72^b^	3.61^a^	3.71^a^	0.09	0.001

*T1: Control group, T2& T3, Probiotics-supplemented groups at 0.25 & 0.5 g/kg, respectively.

LP: Lamina propria, SI: Small Intestine, LI: Large Intestine, TNF-α: Tumor Necrosis Factor- Alpha.

Means with different superscripts in the same row differ significantly (*P* < 0.05).

### Economic evaluation

Groups fed probiotic-supplemented meals had lower overall feeding costs during the research than the control group. Furthermore, the group that received 0.5 g of probiotics had the greatest net profit, economic feed efficiency, and relative economic feed efficiency; the group that received 0.25 g of probiotics had the moderatest of these; and the control group had the lowest (Table 10).

**Table 10:** Effect of dietary treatments on the economic evaluation of the experimental diets.

Item	Treatment*		
	T1	T2	T3
Price (LE/Kg feed)	19.50	19.60	19.70
Total feed cost (LE)	93.80	85.93	77.05
Net profit (LE)	79.05	105.01	144.63
Economic efficiency	0.84	1.22	1.88
Relative economic efficiency	100	145.02	153.60

*T1: Control group, T2& T3, Probiotics-supplemented groups at 0.25 & 0.5 g/kg, respectively.

LE: Egyptian Pound, Net profit = price of kg live body weight - Total feeding cost, Economic efficiency (EE) = net profit / total feeding cost, assuming that the relative economic efficiency of the control diet equals 100.

## Discussion

Probiotics are now increasingly being used as a substitute for antibiotics for growth promotion^36^. Their beneficial impacts on growth parameters stem from various mechanisms, including reducing digestive problems, improving gut health, inhibiting the development of harmful bacteria, decreasing toxin production in the intestine, modulating immunity and antioxidant indicators, lowering blood cholesterol, and regulating animal hematology^37–39^.

The present work demonstrated that adding probiotics to the diets of the growing rabbit at 0.25 and 0.50 g/kg of probiotics improved performance indicators (BW, BWG, FCR, and PI). Notably, when compared to the control group, rabbits fed diets supplemented with 0.50 g/kg probiotics showed an approximate gain of 490 g in body weight, showing a significant improvement in growth performance. Similar observation was reported in rabbit body weight gain, which increased more than 400 g when using a mixture of two probiotics, *Lactobacillus plantarum* and *Bacillus subtilis*^40^ and more than 300 g when using a mixture of three probiotics, *Lactobacillus plantarum*, *Bacillus velezensis*, and *Cyberlindnera fabianii*^41^. Also, the results presented here are supported by previous studies^16,42^ that presented that probiotic dietary supplementation enhanced feed efficiency and growth performance in rabbits. Additionally, the dietary supplementation with probiotics at 1 g/kg improved body weight gain significantly and improved feed efficiency in rabbits^43^. Likewise, supplementing rabbit diets with 400 mg/kg of probiotics enhanced the feed conversion ratio and markedly raised the final body weight (*P* < 0.05)^44^. Moreover, dietary inclusion of a Lactobacillus *plantarum* and *Bacillus subtilis* mix in a growing rabbit’s diet, particularly at a dosage of 0.50 g/kg, plays a significant role in improving feed conversion and body weight gain^40^.

The increase in productive parameters in rabbits fed probiotics may be linked to the role of probiotics in stimulating digestive enzymes, for example amylase, lipase, and protease, which enhance nutrient absorption, metabolism, and digestibility in the gut, as well as in modulating immunity^45^. Improvements in intestinal features, nutritional availability, and growth performance, on the other hand, can be connected to the viable elimination of pathogens and helpful gut microbiota preservation under the influence of probiotics^46^.

Regarding carcass parameters, the current experiment clarified that probiotics significantly increased the weights of the heart, liver, spleen, and lungs. On the other hand, probiotic had a negative effect on abdominal fat percentage. Consistent with our findings, rabbits fed a 400 mg/kg probiotics-enriched diet had significantly larger lung weights^44^. Chowdhury et al. showed that rabbits fed probiotic-supplemented diets had a lower percentage of abdominal fat^47^. The negative impact of probiotics on abdominal fat might stem from activating certain bacteria in the digestive system, which may prevent cholesterol and bile acids absorption^48^. Conversely, other research described that dietary probiotic supplementation had no prominent outcome on the relative weight of kidneys, liver, or carcass percentage in growing rabbits^49,50^.

The health of animals was evaluated with the hematological parameters. In terms of the kind and amount of food that the animals can consume to meet their physiological and metabolic needs, they both reveal and directly reflect the effects of dietary treatments^51^. The positive effect of probiotics on the blood system of rabbits can likely be attributed to the suppression of harmful microflora and the enhancing of the host’s immune system, which may involve increasing or decreasing metabolic activities^39^.

Our hematological results align with a recent study, which revealed a notable increase in erythrocyte counts, hemoglobin, total leukocyte counts, and lymphocyte proportion in the probiotic-treated groups^52^. Other research, however, found no significant differences between rabbits on probiotic-supplemented diets and controls in terms of WBCs, RBCs, and HGB levels^43,53^.

Numerous inconsistencies are found across different probiotic studies, potentially due to factors such as probiotic dosage, animal species, study population characteristics (including breed, weight, age, or gender), the specific microbe strains used, and diet composition.

Blood proteins and globulins are components of the immune system; albumin-based antibodies are the primary protein component of serum, created by hepatic tissues, which play a role in humoral immune responses and may help develop immune organs^54^. Albumin controls the distribution of extracellular fluid and serves as a transporter for various chemicals, including vitamins, fatty acids, hormones, and bilirubin^55^. Our biochemical serum parameter results demonstrated that probiotics have a positive impact on levels of serum total protein and its fraction (albumin and globulin). This result is consistent with a recent study that showed that dietary probiotic supplementation dramatically raised rabbits’ levels of albumin and globulin, raising their total protein levels relative to the control group^56^.

The current data demonstrate that probiotics significantly lower blood lipid levels in rabbits. Their hypocholesterolemic effect appears to involve several mechanisms; these bioactive compounds can bind cholesterol in the intestine, allowing bacteria to use it for producing short-chain fatty acids for their metabolism^57^. Furthermore, some probiotics, such as *Lactobacilli*, can hydrolyze bile salts, blocking their role in cholesterol synthesis. These strains may also suppress the hydroxymethyl-glutaryl-CoA enzyme, which is involved in cholesterol production and converts cholesterol to coprostanol^58^.

Although the probiotics supplementation led to a reduction in the serum liver enzyme activity values (ALT and AST) in the current study, these levels remained within the normal range for rabbits^59^. Serum ALT and AST activities are commonly used to assess hepatic impairment in domestic animals and to detect bile blockage, which indicates a mild to progressive liver disease^60^.

Higher serum levels of triiodothyronine and thyroxine in probiotic-supplemented groups may indicate that the probiotics can increase protein levels and improve dry matter and other nutrients’ digestibility^61^. Similar to our findings, it has been suggested that adding probiotics to the rabbits’ diet raises their serum levels of thyroxine and triiodothyronine^16,56^.

Additionally, the current study showed that dietary probiotic supplementation increased serum immunoglobulin levels, which was consistent with the finding of Alagawany et al.^43^ The modulating effect of probiotics on rabbits’ humoral and cell-mediated immune responses in hot environments has been documented^53^. Furthermore, probiotics can guard animals against pathogen colonization by competing for nutrients and attaching to epithelial sites, boosting the intestinal immune response, and generating antimicrobial bacteriocins. This might also explain how probiotics influence immunity^62^.

Regarding nutrient digestibility coefficients, diets supplemented with probiotics exhibited higher digestibility when compared to the control. The same result was reported with the probiotic Lactobacillus acidophilus, In terms of DM, OM, and CP digestibility was greater than the control^63^. Probiotics enhance digestion through various mechanisms. They regulate intestinal microbiota, affecting bacteria that break down proteins, secrete exoenzymes that aid in protein digestion, stimulate the host’s own digestive enzymes, such as proteases and peptidases, and improve the absorption of amino acids and short peptides by strengthening transport and epithelial capacity^64^.

The gut microbiota influences the digestive system in several ways. It assists the host in breaking down proteins, carbohydrates, and fats, and produces various VFAs and beneficial metabolites through its metabolic, trophic, and protective functions. These substances then affect the local microbial community, helping to maintain the integrity of the gut mucosa and the host’s immune response^65^. In the current trial, probiotic strains have demonstrated the ability to enhance the populations of beneficial microorganisms (mainly *Lactobacillus* spp.) and reduce the presence of potentially harmful bacteria in cecal digesta, such as *Salmonella* spp. The incidence of caecal *lactobacilli* was higher in rabbits that were fed diets fortified with *Lactobacillus acidophilus*. Additionally, *Lactobacillus* species were found to be more effective in inhibiting the invasion and growth of harmful bacteria compared to other bacteria^46^. Furthermore, rabbits given a multi-strain probiotic showed a decrease in intestinal coliform populations and an increase in cecal *lactobacilli* populations^4,66^.

Our histological data showed that supplementation with two levels of probiotics significantly improved histological parameters of the rabbit’s SI, especially inducing villus length, epithelial thickness, and CD.

These morphometrical modifications are important indicators of enhanced SI function. Our data agree with previous studies that have reported probiotics enhance intestinal villi length, CD, and epithelial thickness, thereby increasing the surface area of absorption and preventing the penetration of harmful materials, which improves the animal’s performance and immunity^16,67–71^. Histochemical assessment of the SI using PAS stain revealed an increase in mucus in the groups receiving the probiotics. Following probiotic food supplementation, several studies found an increase in goblet cells in the small intestine, which forms the mucous layer of the intestine that acts as a key barrier to protect the intestine^72–74^.

In accordance with large intestine histological assessment, we observed improvement in epithelial thickness and CD after probiotics administration, particularly in high doses. Previous studies have highlighted the role of probiotics in enhancing the LI lining epithelium and crypts, describing probiotics as important players in intestinal cell renewal and the restoration of the intestinal barrier through the regulation of cell apoptosis and proliferation^75–77^.

Our data revealed non-significant differences between groups in the diameter of hepatocytes and hepatocyte nuclei. This is an interesting observation due to the cholesterol level being low and the liver weight being increased. Probiotics can decrease cholesterol level through decreasing its absorption and increasing its excretion depending on direct intestinal assimilation of cholesterol. This reduces the systemic lipid load without causing large hepatocyte changes^78–80^. However, histochemical data indicated that PAS stain intensity increased in the livers of rabbits fed probiotic supplements, particularly with the high dose. Previous studies have reported that probiotic supplementation increases PAS positivity in the liver, attributing this to increased glycogen storage in hepatocytes^81–83^. Moreover, a rise in the amount of glycogen is linked to an increase in the weight of the liver, which is also related to an increase in body weight^84,85^.

In contrast, histomorphometric data from the kidney supplemented with a high dose of probiotic mixture showed an improvement in the diameter of both renal corpuscles and glomeruli. Probiotics have been previously described as having the potential to improve kidney function and exert a protective effect on the kidneys^86–88^. Also, our results detected an increase in CMFs diameter in groups taking both concentrations of the probiotic mixture. Previous studies have described the role of probiotics in enhancing cardiac performance, reducing myocardial injury, and improving cardiovascular health^89,90^.

To estimate the immunomodulatory effects of probiotics dietary supplementation, we estimated TNF-α immunohistochemical expression. TNF-α acts as a pro-inflammatory cytokine and plays a crucial role in regulating immunity^91,92^. Our data revealed that TNF-α expression in the intestine increases significantly with probiotics supplementation, with the high dose being most significant in the SI and the low dose in the LI. The increase in TNF-α may be related to immune stimulation in healthy mucosa. Previous studies have found that probiotics induce immune cells and pro-inflammatory cytokines, considering their role in the induction of gut mucosal immunity^93–95^.

To investigate how probiotic dietary supplements affect the intestine’s neuroendocrine characteristics, we evaluated synaptophysin immunohistochemical expression. Synaptophysin is used as a neuroendocrine marker in different organs, including the gastrointestinal tract^96,97^. We found a significant increase in expression with both doses of probiotic dietary supplementation in the LI, with non-significant differences between the two doses. Nevertheless, the SI showed no apparent differences across the groups. This data reflected the neuroendocrine modulatory effect of probiotics in the LI. Neuroendocrine cells play a crucial role in regulating gut secretory activity, motility, and immune response^98,99^. In addition, the neuroendocrine cells of the gastrointestinal tract and the microbiota both influence metabolism and maintain homeostasis, and are interrelated^100^.

The economic assessment of the rabbit diets showed that groups fed probiotic-supplemented diets had higher final BW, which correlated with increased net profit and better economic efficiency. Furthermore, the probiotic-fed groups exhibited reduced feed intake, resulting in lower feed costs. These results align with previous research indicating that dietary probiotic supplementation affects economic outcomes. The observed improvements over the control group likely stem from increases in live body weight, weight gain, and improved feed conversion ratios in the treated groups^16,49^.

In spite of the valuable results of this study regarding the probiotic impact on rabbit nutrition, some limitations should be considered. Research integrating growth performance, digestive efficiency, blood parameters, cecal microbiota, histological and immunohistochemical evaluations is still limited; this makes direct comparison difficult. Future experiments with different rabbit breeds and at different physiological and environmental conditions are necessary to do. Additionally, the use of advanced molecular, histological, and immunohistochemical approaches is essential in feeding experiments to clarify the results and explain the biological mechanisms.

## Conclusion

Adding two levels of probiotics, 0.25 g/kg and 0.50 g/kg of *Lactobacillus plantarum* and *Lactobacillus acidophilus*, to the basal diet of growing rabbits was associated with improvements in growth performance. It also contributed to reductions in abdominal fat and blood lipid levels and was linked to favorable changes in hematological and serum biochemical parameters, increased nutrient digestibility, promoted the growth of beneficial bacteria, and suppressed the proliferation of harmful bacteria in the cecum. Additionally, it may have supported neuroendocrine function in the intestine, boosted immunity, and improved several intestinal morphological features, including villus length, epithelial thickness, and CD. The supplementation also showed positive effects on the histomorphometry of renal, hepatic, and cardiac tissues and improved the economic value of the diet.

## Data Availability

The datasets utilized and/or examined in the present investigation can be obtained from the corresponding author upon a reasonable request.
